# Animal-Based Factors Prior to Infection Predict Histological Disease Outcome in Porcine Reproductive and Respiratory Syndrome Virus- and *Actinobacillus pleuropneumoniae*-Infected Pigs

**DOI:** 10.3389/fvets.2021.742877

**Published:** 2021-11-17

**Authors:** Ingrid D. E. van Dixhoorn, Dennis E. te Beest, Jantina E. Bolhuis, Hendrik K. Parmentier, Bas Kemp, Simon van Mourik, Norbert Stockhofe-Zurwieden, Cornelis G. van Reenen, Johanna M. J. Rebel

**Affiliations:** ^1^Wageningen Livestock Research, Department of Animal Health and Welfare, Wageningen, Netherlands; ^2^Biometris, Wageningen University & Research, Wageningen, Netherlands; ^3^Adaptation Physiology Group, Wageningen University & Research, Wageningen, Netherlands; ^4^Farm Technology Group, Wageningen University & Research, Wageningen, Netherlands; ^5^Wageningen Bio-Veterinary Research, Lelystad, Netherlands

**Keywords:** resilience indicators, porcine respiratory disease, PRRSV, *Actinobacillus pleuropneumoniae*, coping strategy, enriched housing, disease severity, animal-based factors

## Abstract

A large variety of clinical manifestation in individual pigs occurs after infection with pathogens involved in porcine respiratory disease complex (PRDC). Some pigs are less prone to develop respiratory disease symptoms. The variation in clinical impact after infection and the recovery capacity of an individual animal are measures of its resilience. In this paper, we examined which ones of a range of animal-based factors (rectal temperature, body weight, skin lesion scores, behavior, natural antibody serum levels, serum levels of white blood cells, and type of T and granulocyte subsets) when measured prior to infection are related to disease severity. These animal-based factors and the interaction with housing regimen of the piglets (conventional or enriched) were modeled using linear regression to predict disease severity using a dataset acquired from a previous study using a well-established experimental coinfection model of porcine reproductive and respiratory syndrome virus (PRRSV) and *Actinobacillus pleuropneumoniae*. Both PRRSV and *A. pleuropneumoniae* are often involved in PRDC. Histological lung lesion score of each animal was used as a measure for PRDC severity after infection. Prior to infection, higher serum levels of lymphocytes (CD3^+^), naïve T helper (CD3^+^CD4^+^CD8^−^), CD8^+^ (as well as higher relative levels of CD8^+^), and memory T helper (CD3^+^CD4^+^CD8^+^) cells and higher *relative* levels of granulocytes (CD172^a^) were related to reduced disease severity in both housing systems. Raised serum concentrations of natural IgM antibodies binding to keyhole limpet hemocyanin (KLH) were also related to reduced disease severity after infection. Increased levels of skin lesions at the central body part (after weaning and before infection) were related to increased disease severity in conventional housing systems only. High resisters showed a lower histological lung lesion score, which appeared unrelated to sex. Body temperature, behavior, and growth prior to infections were influenced by housing regimen but could not explain the variation in lung lesion scores after infection. Raised basal lymphocyte counts and lower skin lesion scores are related to reduced disease severity independent of or dependent on housing system, respectively. In conclusion, our study identifies intrinsic animal-based measures using linear regression analysis that predicts resilience to infections in pigs.

## Introduction

Porcine respiratory disease complex (PRDC) is an example of a typical polymicrobial production disease that can cause major concerns for animal welfare as well as economic losses in the pig industry worldwide (1). Pathogenesis of these multifactorial polymicrobial diseases involves infectious factors of different pathogens (both viral and bacterial) and noninfectious factors. There are a variety of pathogens associated with PRDC, classified as either primary or secondary agents. These pathogens include porcine reproductive and respiratory syndrome virus (PRRSV), swine influenza virus (SIV), porcine circovirus type 2 (PCV2), *Mycoplasma hyopneumoniae, Pasteurella multocida*, and *Actinobacillus pleuropneumoniae* (1). Secondary agents are opportunistic bacteria that will invade the damaged lung following previous infection with a primary agent (often PRRSV) (2). However, in a highly complex disease situation, several primary agents can act together and subsequently produce a population disease picture that is very difficult to unravel (2).

Noninfectious factors are important and can play a role in the initiation and disease outcome (1) and consist of the following: 1) environmental factors, 2) type of production system and management, and 3) pig specific factors (genetics, sex, age, coping strategy, immune status, and resilience state of the pig). Resilience state of pigs can be defined as the capacity to withstand perturbations such as infections and can be quantified afterwards by measuring variation of clinical impact and recovery capacity after infection of an individual animal in terms of severity and duration of symptoms (3). Thus, the variation in severity of symptoms between individual pigs caused by infection of these polymicrobials is not only due to the variation of pathogens and their virulence, but pigs kept under similar circumstances will show individual variation in disease manifestation as well (4). The individual variation in clinical outcome or resilience after infection has for some part previously been explained by differences in genetic background, age, and also the immune status (5–8). Especially maternal antibody levels have been shown to be of influence on the clinical manifestation in individual pigs (9). Clinical cough symptoms and body temperature at the start of infection have been found to be of significance as indicators for the severity of symptoms after *Escherichia coli* endotoxin and *P. multocida* challenge in pigs (10). Animal-based specific factors such as age, genetic background, and sex may be of influence on disease severity, but which other additional animal-based factors at time of infections will predict resilience is still largely unknown (11). How the interaction between animal-based factors and housing or environmental factors prior to or during infections affects disease severity remains to be elucidated. High resilient animals or vulnerable animals can be recognized more precisely when more knowledge is available about the interplay between individual animal-based factors in combination with environmental and social circumstances. This will enable early preventive intervention strategies and/or methodological and conceptual tools to improve predictive value of disease outcome in the future (12) in complex diseases such as PRDC. Embedding animal-based indicators in model-based approaches can possibly be used in prevention and control strategies at the individual operational level (e.g., extra temporary extra supportive attention or changes in diet, housing, or climate control to specific animals or groups) or at the strategic management level (e.g., changing management measures, housing condition, and breeding strategy).

This study focused on relations between animal-based measures *before* infection and the severity in terms of pathological presentation of PRDC-related lung lesions *after* infection. A defined set of animal-based measures was tested and consisted of coping strategy, sex, rectal temperature, weight/growth, activity, behavior, skin lesions, white blood cell (WBC) counts, and phenotypical T and granulocyte differentiation and serum natural antibody (NAb) levels. Interaction with type of housing system was tested (enriched and low stocking density or conventional housing system). A dataset originating from an experiment with 28 pigs was used in a well-established experimental coinfection PRDC model of PRRSV and *A. pleuropneumoniae* (13).

## Materials and Methods

### Animals, Experimental Design, and Treatments

A dataset of parameters of 28 piglets was used originating from an experiment described earlier (13, 14). For the experiment, the established principles of laboratory animal use and the EU and Dutch laws related to animal experiments were adhered to in this study. The Wageningen University Animal Care and Use Committee (Lelystad Department) approved the experiment under number 2013181.

The 28 piglets were a selection of the offspring (56 male and female piglets) of eight multiparous sows obtained from a PRRSV- and *A. pleuropneumoniae*-free herd. From the first day of life onwards, half of the pigs were housed in four conventional 5-m^2^ pens (seven pigs per pen) with 100% slatted floor and a 100 × 45 cm solid rubber floor mat [conventional housed pigs (CH pigs)]. The other half of the pigs were housed in four enriched pens (10 m^2^, seven pigs per pen) with partly slatted (40%) and partly solid (60%) floors [enriched housed pigs (EH pigs)]. Enrichment consisted of two chains with plastic blocks in both housing conditions. In the enriched pen, two jute bags and branches of a broom were provided as well as rooting substrate consisting of straw, peat, and wood shavings [see Van Dixhoorn et al. (13) for details]. Social enrichment was applied to the enriched pens from 13 days of age until weaning, by removing the panels between two adjacent enriched pens until weaning. Each pen was cleaned daily, and enrichment materials and food were γ-irradiated (9-kGy irradiation; Synergy Health Ede BV, Ede, Netherlands). Each pen had two drinking nozzles, one for the sow and one for the piglets. Sows were fed a standard commercial diet twice daily. From 3 days of age, the piglets received solid food *ad libitum*. Lights in the pens were on between 6:00 a.m. and 6:00 p.m., and the temperature gradually decreased from 25 to 22°C the week before weaning. After weaning at the age of 31 days, 14 piglets were selected per housing treatment. The selection was made taking sex, coping strategy, and weight into account in order to obtain piglets with comparable features in each experimental subgroup. All piglets were equally mixed, and four new groups (two enriched and two conventional) of seven pigs each were formed and balanced for sex, coping strategy, and weight. Coping strategy was assessed at the age of 17 days by performing a backtest (15). During the backtest, piglets were held in supine position for 1 min; and the number of struggles, latency to first struggle, number of vocalizations, and latency to the first vocalization were recorded. Pigs were classified into “high-resisters” (HR) and “low-resisters” (LR) as described previously by Bolhuis et al. (16). This resulted in characterization of each pig by mother (genetic background), pen, sex, coping strategy, and housing treatment. These pig specific data were used as experimental variables. Dataset details are summarized in Table 1.

**Table 1:** Dataset details: experimental setup.

**Pig number**	**Pen**	**Sex**	**Coping strategy**	**Housing**
14	2	Male 6	High resisters 2	Conventional
		Female 8	Low resisters 12	
14	2	Male 6	High resisters 3	Enriched
		Female 8	Low resisters 11	

At the age of 44 days, CH and EH pigs were intranasally infected with 1.5 ml of inoculum containing 5 log_10_ 50% tissue culture infectious dose (TCID) of the mild-virulent European PRRSV serotype 1 strain LV-Ter Huurne [described in Van Dixhoorn et al. (13)]. This treatment was followed by an aerosol infection at day 52 (ID8) with *A. pleuropneumoniae* serotype 2. Groups of two to three pigs were simultaneously exposed in a stainless-steel aerosol chamber (110 × 45 × 90 cm). An amount of 5 ml of the inoculum suspension was administered during a period of 20 min using the aerosol nebulizer Aeroneb Pro (EMKA Technologies, Paris, France). The procedures have previously been described (17). On day 55, the pigs were euthanized by injection of pentobarbital (Euthasol 40%, AST Farma, Oudewater, Netherlands) in the auricular vein while being restrained and thereafter exsanguinated, followed by necropsy.

### Measurement of Disease Severity: Histological Assessment of the Lungs

**Figure 1:**
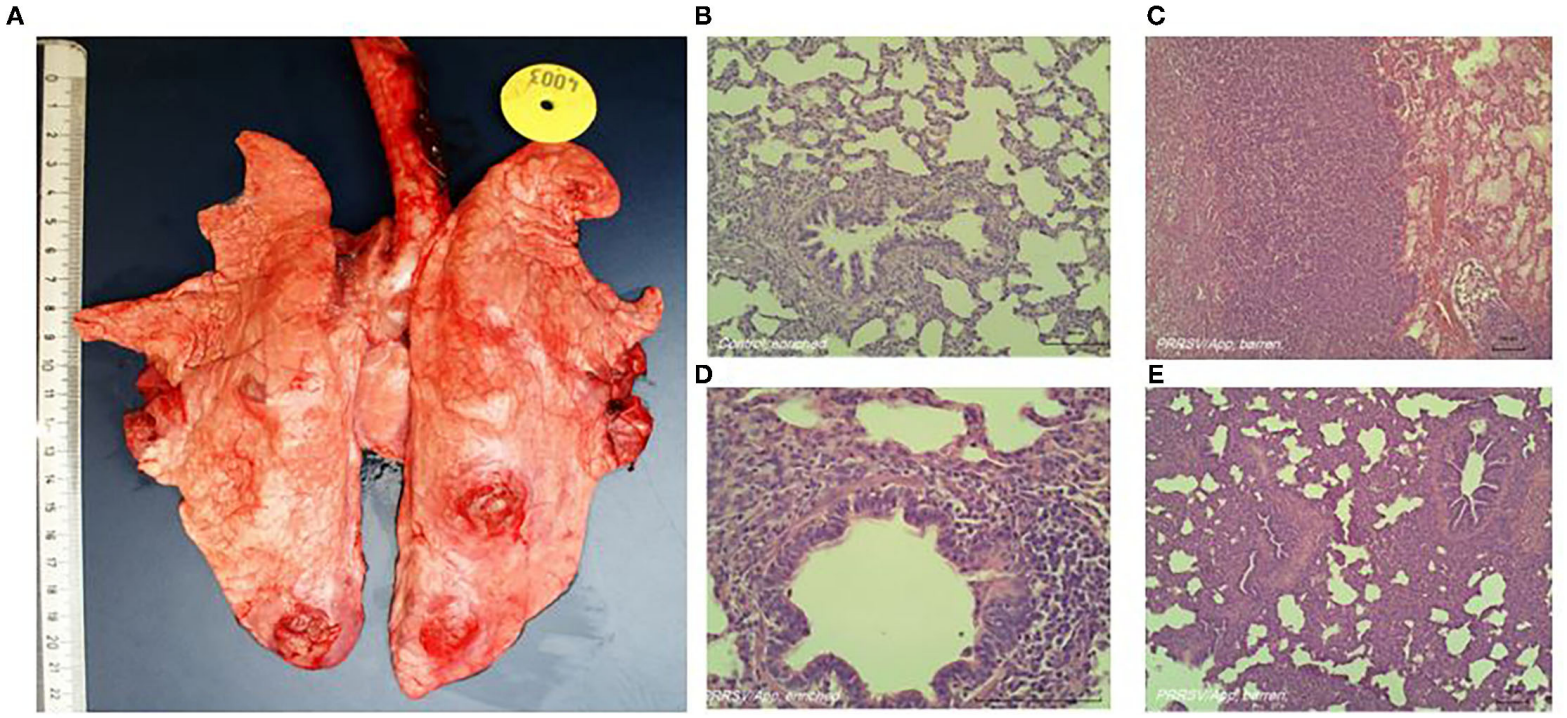
Representative macroscopic **(A)** and histologic **(B–E)** lung lesions caused by coinfection of porcine reproductive and respiratory syndrome virus (PRRSV) and *Actinobacillus pleuropneumoniae*. **(B)** Absence of histological alterations; healthy lungs in non-infected animals. **(C)** Diffuse severe alterations (interstitial or catarrhal pneumonia, infiltrations in alveolar septa and peribronchial or perivascular area, pleuritis). **(D)** Close-up of (mild) infiltrations of perivascular and peribronchial area. **(E)** Diffuse severe alterations (interstitial or catarrhal pneumonia, infiltrations in alveolar septa and peribronchial or perivascular area, pleuritis).

Typical lung lesions caused by the coinfection are shown in Figure 1A. The histological score of the lungs, obtained during necropsy, was used as indication for severity of coinfection. Six tissue samples per pig from predefined locations in the lungs (cranial, cardiac, and caudal lobes of the left and right lobes) were formalin-fixed, processed, and embedded in paraffin. Tissue sections were stained with H&E; and a semiquantitative, patho-histological assessment of H&E-stained slides encompassed the extent of pneumonia throughout the predefined locations in the lungs. Patho-histological assessment included four features: 1) the presence of focal or diffuse alterations with interstitial or catarrhal pneumonia or atelectasis, 2) the extent of infiltration of the alveolar septa with mononuclear cells, 3) the extent of infiltration of mononuclear cells in the perivascular/peribronchial area, and 4) pleuritis. Examples of typical histological lesions are shown in Figures 1B–E. A histological score of 0 to 3 was used to describe the severity of changes per feature (i.e., 0 = no findings; 1 = mild focal manifestation; 2 = moderate, multifocal manifestation, or diffuse manifestation; 3 = severe diffuse manifestation). During a histological examination, the pathologist was unaware of housing or coping treatment. The scores from all six slides per lung were added to obtain an overall histology score, which could add up to a maximum of 72 points per pig (six slides per pig, four histological features, and maximum score of three per feature).

### Measurements of Animal-Based Factors

Measurements prior to infection were used as possible explanatory predictive variables and consisted of rectal temperature, body weight at different time points, behavioral assessment, skin lesion scores, WBC counts, and concentrations of serum natural antibodies.

Rectal temperature was measured twice at days 40 and 44 of age prior to infection. Microlife digital thermometers with a resolution of 0.1°C were used. Thermometers were calibrated prior to the experiment. Body weight of the piglets was measured weekly from the day of parturition until the end of the experiment. Difference in body weight between the measurement after weaning at day 44 and before weaning was also calculated.

Frequencies of the behaviors listed in the ethogram [Table 2, adapted from Camerlink et al. (18)] were recorded four times, at days 30, 32, 38, and 40. On each day, all pens were observed for four 10-min periods, twice in the morning (between 8:00 and 11:00 a.m.) and twice in the afternoon (between 1:00 and 4:00 p.m.) in an order balanced for housing. Behaviors were expressed as frequencies per pig per 10 min. A new bout was scored when the pig stopped the behavior for more than 2 s. On the same days, skin lesions at the front (head, neck shoulders, and front legs), middle (flanks and back), and rear (rump, hind legs, and tail) were counted and categorized as a proxy for aggressive behavior (19). For each body region, the number and severity of lesions were differentiated using scores from 0 to 4, as follows: 0, no lesions; 1, <5 superficial lesions; 2, 5–10 superficial lesions or <5 deep lesions; 3, 10–15 superficial lesions or 5–10 deep lesions; and 4, >15 superficial lesions or >10 deep lesions. Lesion scores were averaged per day.

**Table 2:** Ethogram.

**Behavior**	**Description**
Social behavior	Touching or sniffing any body part of a pen mate
Aggression	Uni- or bilateral fighting by chasing, head knocking (with or without biting), and/or pushing
Manipulate pig	Nibbling, sucking, or chewing on any body part of piglet or sow, or belly nosing
Manipulate pen	Nibbling, sucking, or chewing on pen components
Playing	Fast running around the pen (galloping), rolling, and shaking objects
Mounting	Standing on hind legs with front legs on pen mate

Blood samples taken by jugular vein puncture when piglets were 44 days of age were used to establish total WBC count and phenotyping of WBC (serum and EDTA blood, respectively). A differential count of lymphocytes, granulocytes, and monocytes was assessed using a hematology analyzer (blood cell counter Sysmex pocH-100iV diff, Kobe, Japan) as described in Van Dixhoorn et al. (13). Freshly isolated heparinized blood samples (100 μl) were used to quantify different phenotypes of WBCs followed by fluorescence-activated cell sorting (FACS) analysis (FACS Lysing Solutions BD Biosciences, San Jose, CA, USA) as described in Van Dixhoorn et al. (13). NK cells (CD3^−^CD4^+^CD8^+^), naïve T helper cells (CD3^+^CD4^+^CD8^−^), memory T helper cells (CD3^+^CD4^+^CD8^+^), and CD8^+^ cells were identified and presented as absolute cell counts. Relative levels of different types of WBC were then calculated as percentage of total WBC. Both absolute cell counts and relative levels of different types of WBC were tested as possible explanatory predictive variables.

**Figure 2:**
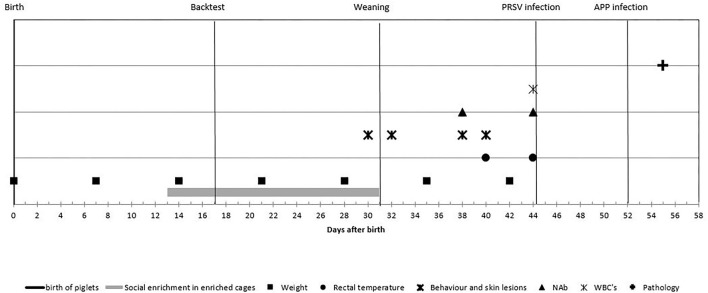
Experimental process and timeline of the measurements: piglets were observed from birth until 55 days of age. Enriched housed pigs experienced social enrichment from day 13 until weaning; coping strategy was assessed at day 17. Piglets were weaned at day 31 and infected with porcine reproductive and respiratory syndrome virus (PRRSV) at day 44 and with *Actinobacillus pleuropneumoniae* at day 52 and euthanized at day 55. Body weight was measured weekly; rectal temperature was measured at days 40 and 44; behavior and skin lesions were assessed at days 30, 32, 38, and 40. Natural antibodies (NAbs) were measured at days 38 and 44; white blood cell (WBC) at day 44 and histological lung lesion scores were assessed after day 55.

Blood samples taken by jugular vein puncture when piglets were 38 and 44 days of age were used to establish levels of NAb binding to keyhole limpet hemocyanin (KLH) or phosphoryl-conjugated bovine serum albumin (PC-BSA, Sigma-Aldrich, St. Louis, MO, USA) and natural antibodies binding self-antigen (NAAb) to myelin basic protein (MBP, Santa Cruz Biotechnology, Santa Cruz, CA, USA) according to procedures described by Luo et al. (14). In short, titers of IgM and IgG antibodies binding (KLH, MBP, or PC-BSA) were determined by a two-step indirect ELISA. Each absorbance was expressed relatively to the absorbance of a standard positive control serum sample, and antibody titers were expressed as log2 values of dilutions with extinction closest to 50% of Emax, where Emax represents the highest mean extinction of a standard positive serum present on every microtiter plate. Experimental process and timeline are visualized in Figure 2.

### Statistical Analysis

**Figure 3:**
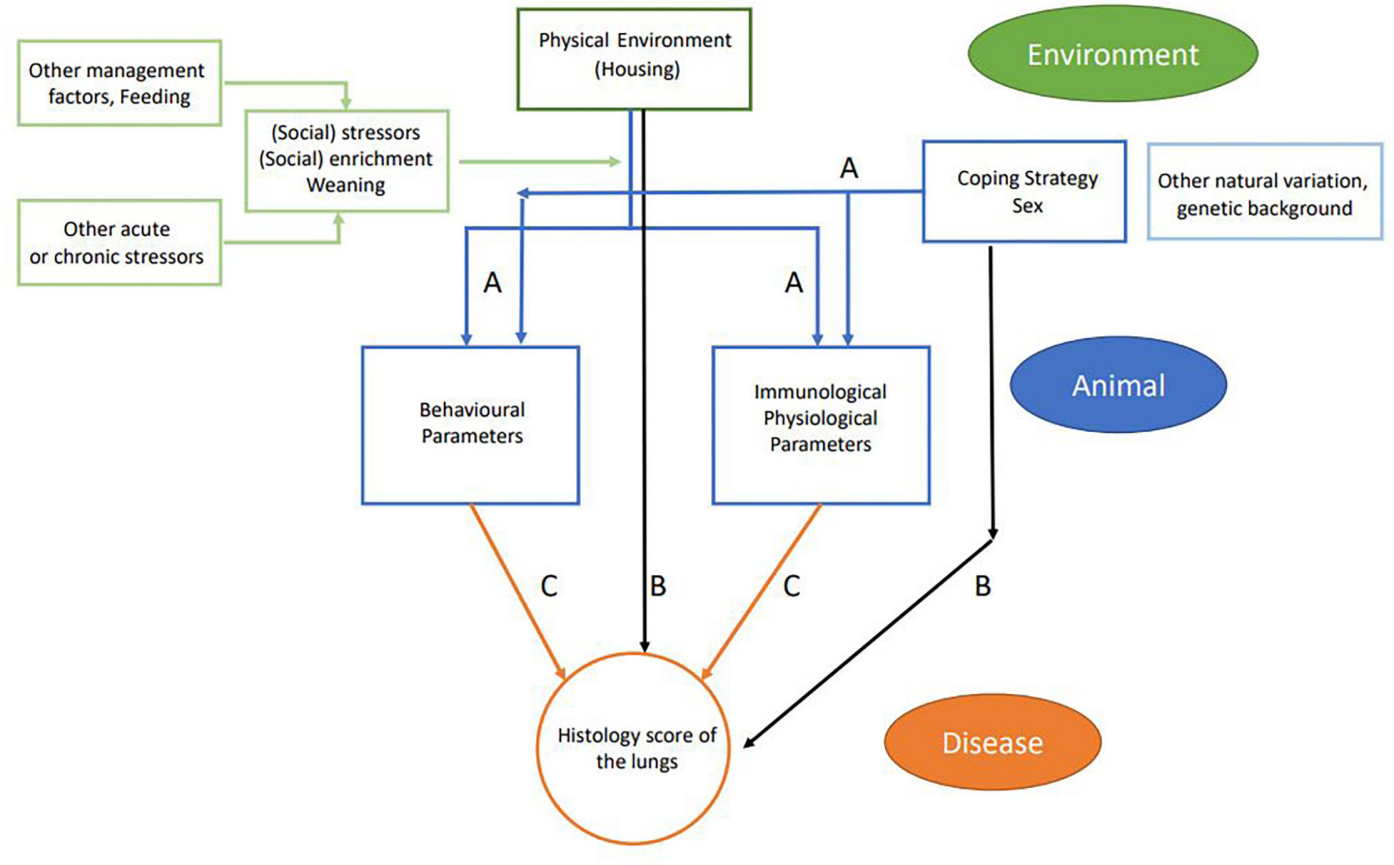
Schematic flowchart of linkage between farm management practices (environment and management factors in green), animal-based measures (in blue), and disease outcome (in orange). Arrows indicate the different relations that were tested: blue arrows **(A)** relate to experimental variables (housing, coping, and sex) and explanatory variables; black arrows **(B)** relate to experimental variables (housing coping and sex) and disease outcome; and orange arrows **(C)** relate to explanatory variables and disease outcome including housing, coping, and interaction housing × explanatory interaction.

The usage of the variables in the statistical models is explained in Table 3 and consisted of four parts. First, the effect of the experimental variables (housing, coping, and sex) on the explanatory variables was tested with a linear mixed model. Per explanatory variable, we fitted a model with the explanatory variable as the dependent variables; the experimental variables as the independent variables; and pen as a random effect (corresponding to arrows A in Figure 3). Second, we explored the effect of experimental variables on the histology score. This was also done with a linear mixed model that included histology score as the dependent variable, the experimental variables (housing, coping, and sex) as the independent variables, and pen as a random effect (corresponding to arrows B in Figure 3).

**Table 3:** Overview of the used variables and disease outcome.

**Experimental setup variables**	**Explanatory variables**	**Dependent variable clinical severity**
	Rectal temperature	Histological score
Pen (random)	Body weight	
Coping Strategy	Behavior	
Sex	Skin lesions	
Housing	WBC	
	NAb	

*WBC, white blood cell; NAb, natural antibody*.

In the third step, we explored the effect of explanatory variables on the histology score (corresponding to arrows C in Figure 3). In this model, histology score was used as the dependent variable. Each explanatory variable was analyzed separately as an independent variable on top of a model that contained housing, coping, and pen (random effect). Sex was not included, as we did not find a relation between sex and histology score in the previous analysis. Per model, we tested for an interaction between housing and the explanatory variable. The interactions between coping and the explanatory variable could not be estimated due to the small number of high resisters. All models were fitted using R package lme4, and *p*-values were calculated using package lmerTest. In parts 1 and 3 of the analysis, results were adjusted for multiple testing. In this adjustment, we calculated the false discovery rate (FDR) per variable set with the Benjamini–Hochberg correction.

In the last step, we explored if the model using the histology score as the dependent variable could be improved by including more explanatory variables (multivariable approach). With the use of the base model that included housing, coping, and pen (random effect), explanatory variables were added using forward selection based on improving the Akaike information criterion (AIC), to determine which model fitted best. As an additional criterion in this procedure, the sign of an effect size should not change direction when a new variable is included, suggestive of co-linearity of data.

## Results

### Effect of Experimental Setup Variables (Housing, Coping Strategy, and Sex) on Explanatory Variables Assessed Prior to Infection

First, the effect of housing on explanatory variables that were measured prior to infection was tested (corresponding to arrows A in Figure 3). In conventional housed pigs, the temperature 4 days prior to infections was higher, more skin lesions were apparent at the rear of the body after weaning, and pigs showed more mounting behavior. Overall absolute WBC and lymphocytes counts were lower in conventional housed pigs prior to infection.

**Figure 4:**
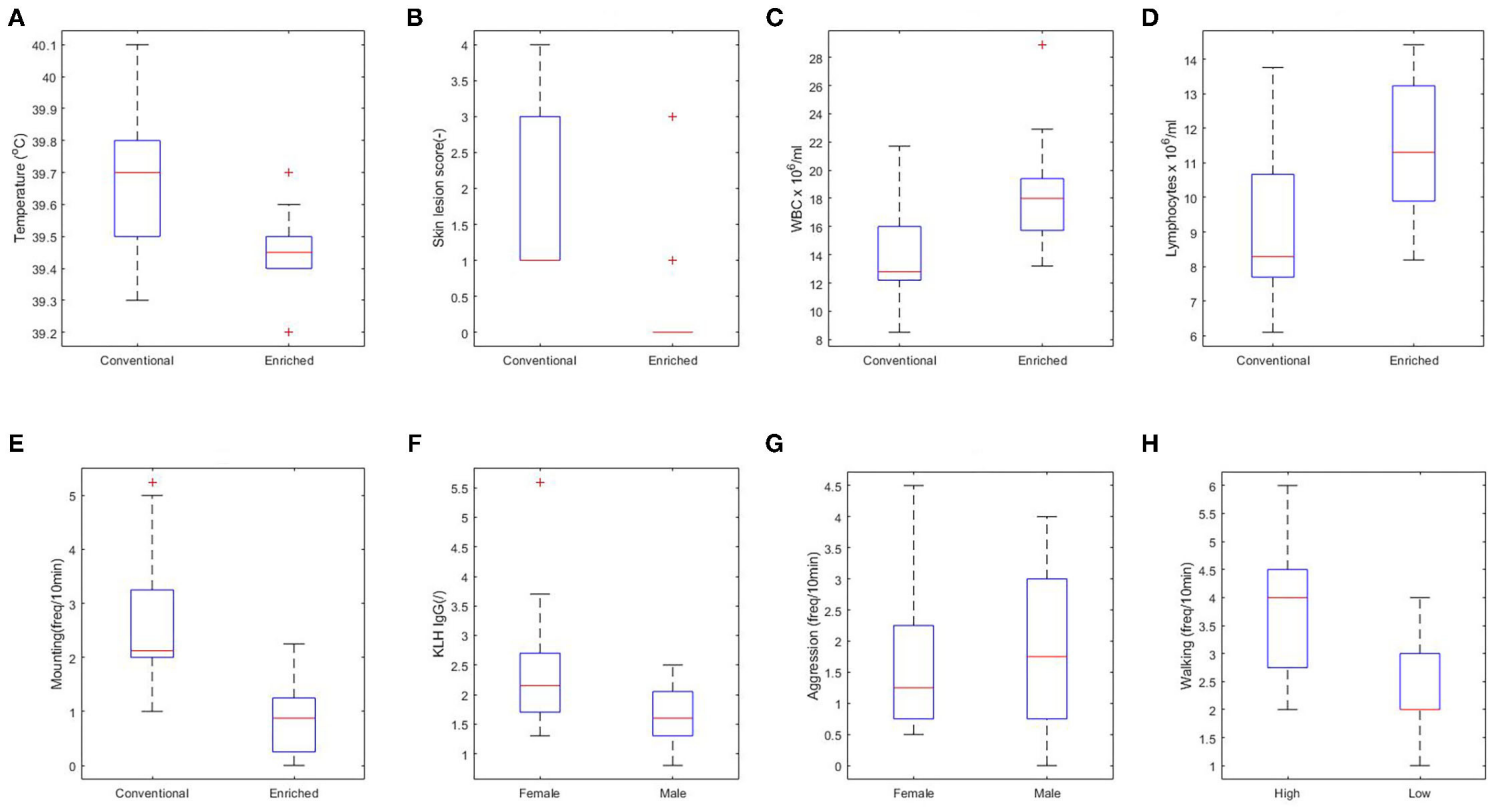
Significant relations between housing **(A–E)**, sex **(F,G)**, and coping strategy (H) and explanatory variables. **(A)** Rectal body temperature at day 40 (*p* < 0.01). **(B)** Skin lesions at the rear part of the body at day 38 (*p* < 0.001). **(C)** White blood cell (WBC) at day 44 (*p* < 0.05). **(D)** Lymphocytes at day 44 (*p* < 0.05). **(E)** Overall mounting behavior (*p* < 0.001). **(F)** Keyhole limpet hemocyanin (KLH) IgG at day 38 (*p* < 0.01). **(G)** Overall aggressive behavior (*p* = 0.06). **(H)** Walking activity (*p* < 0.01).

Independent of housing, female piglets had higher levels of KLH-binding IgG antibodies at day 38 and lower aggression scores during the complete period prior to infection than had the males. The separate analysis of coping strategy effect showed more walking behavior prior to infection by high resisters as compared with the low resisters (Figure 4).

### Effects of Experimental Variables (Housing, Coping Strategy, and Sex) on Disease Severity

**Figure 5:**
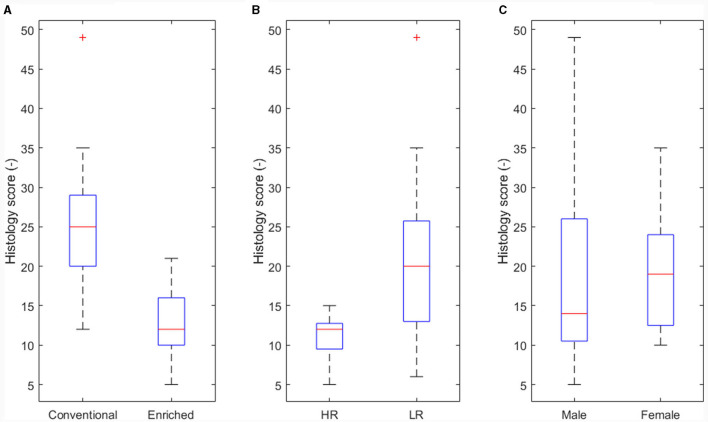
Main effect of experimental variables on histology score. **(A)** Housing (*p* < 0.05). **(B)** Coping strategy (*p* < 0.05). **(C)** Sex not significant (NS).

The effect of housing on disease severity (corresponding to arrows B in Figure 3) was also presented earlier (13), showing more severe histology scores in conventional housed pigs (Figure 5A, p < 0.05).

An effect of coping strategy was also seen in histology score, where the high resisters showed lower histological scores (p < 0.05, Figure 5B) than did the low resisters. An effect of sex on disease severity was not found (Figure 5C).

### Effects of Explanatory Variables on Histology Score

All explanatory variables were tested to predict histology score as disease severity read out (corresponding to arrows C in Figure 3). The results with histology as response variable and no interaction effect with housing are presented in Table 4 (only the significant explanatory variables with *p* < 0.05 and FDR < 0.1 are shown). The results with significant interaction effect with housing are shown in Table 5. Temperature and weight variables did not relate to histology scores.

**Table 4:** Significant explanatory variables for histology score as response variable with no interaction with housing.

**Explanatory variable (day 44)**	**Coefficient**	***p*** **-Value**	**FDR**	**R^2^**
CD8^+^ cells	−9.47014	<0.05	0.06	0.07
Lymphocytes	−1.62288	<0.01	0.01	0.11
Memory T helper cells	−88.5637	<0.01	0.01	0.07
Naïve T helper cells	−10.786	<0.01	0.01	0.10
Relative granulocytes	38.27377	<0.01	0.03	0.08
Relative Lymphocytes	−48.2953	<0.05	0.03	0.08
Relative naïve T helper cells	−244.9	<0.01	0.02	0.08

*Variables with FDR < 0.10 are presented in the table. Absolute counts and relative count as percentage of WBCs are depicted. FDR, false discovery rate; WBC, white blood cell*.

**Table 5:** Interaction effects with type of housing and explanatory variables with histology score as response variable.

**Explanatory variable**	**Housing**	**Coefficient**	***p*** **-Value**
Relative CD8^+^ cells	Conventional	−319.202	<0.01
KLH binding IgM, day 40	Conventional	−6.23362	<0.001
Skin lesions central, day 40	Conventional	7.229481	<0.001

*Variables with p < 0.05 and FDR < 0.10 are presented. KLH, keyhole limpet hemocyanin; FDR, false discovery rate*.

Lower absolute cell counts and relative levels of lymphocytes and T helper cells, and lower absolute cell counts of CD8^+^ cells and memory T helper cells prior to infection were related to higher histology score. This effect was independent of the type of housing. Higher relative level of granulocytes prior to infection was related to higher histology score. No other variables showed a relation with histology score independent of housing regimens.

When the interaction housing × response variable was found significant (p < 0.05), significance was tested for the two different housing conditions. A lower relative level of CD8^+^ cells and lower KLH IgM levels before infections were related to higher histology scores in the lungs in conventional housed pigs only. Higher skin lesion scores after weaning at the center part of the body were related to higher histology scores in the conventional housed pigs (Table 5).

### Effects of Multiple Explanatory Variables on Disease Severity

The absolute cell counts and relative levels of WBC differentiations were tested separately. For the absolute cell counts, the histology score could be explained by the base model with the lowest AIC criterion using the absolute level of memory T helper cells, including pen as random effect and coping and housing as fixed effects:


$$\begin{array}{l} \text{histology score}\sim \text{coping + }(\text{random pen})\text{ + housing }\\ \text{ + memory T helper cells} \end{array}$$

Then it was tested if AIC was improved by adding other explanatory variables (Table 6). In each step, another variable was added to the equation. Adding lymphocytes and CD8^+^ cells to the equation slightly improved the AIC, but with the addition of naïve T helper cells, the AIC was the lowest (Table 6). Then it was tested if the addition of a third variable to the same model could improve the AIC. The inclusion of CD8^+^ cells to the statistical model changed direction of the sign of the effect and is therefore probably attributed to co-linearity of input variables. Higher histological lung scores were best explained by both lower levels of memory and naïve T helper cells. So the model with the lowest AIC was as follows:


$$\begin{array}{l} \text{histology score}\sim \text{coping + }(\text{random pen})\text{ + housing }\\ \text{ + memory T helper cells }\\ \text{ + naïve T helper cells} \end{array}$$

**Table 6:** Multivariable approach results with absolute numbers of different white blood cells.

**Base model absolute numbers**	**Adding**	**Adding**	**AIC**
Memory T helper cells	–	–	147
Memory T helper cells	Lymphocytes	–	145
Memory T helper cells	CD8^+^ cells	–	143
Memory T helper cells	Naïve T helper cells	–	141
Memory T helper cells	Naïve T helper cells	Lymphocytes	141
Memory T helper cells	Naïve T helper cells	CD8^+^ cells	137^*^

This same procedure was performed with relative levels of different WBC, resulting in the lowest AIC reached when naïve T helper cells, memory T helper cells, and lymphocytes were used in the model. Again, CD8^+^ cells reduced AIC, but the change in direction of the effect suggests co-linearity (Table 7).

**Table 7:** Multivariable approach results with relative numbers of different white blood cells.

**Base model relative numbers**	**Adding**	**Adding**	**AIC**
Naïve T helper cells	–	–	143
Naïve T helper cells	Lymphocytes	–	137
Naïve T helper cells	Memory T cells	–	131
Naïve T helper cells	CD8^+^	–	133^*^
Naïve T helper cells	Granulocytes	Lymphocytes	137
Naïve T helper cells	Memory T cells	CD8^+^ cells	124
Naïve T helper cells	Memory T cells	Granulocytes	125

*AIC, Akaike information criterion*.

* *Inclusion of CD8^+^ cells changed direction of the sign of the effect*.

Final model with relative numbers of WBC:


$$\begin{array}{l} \text{histology score}\sim \text{coping + }(\text{random pen})\text{ + housing }\\ \text{ + memory T helper cells }\\ \text{ + naïve T helper cells + lymphocytes} \end{array}$$

## Discussion

Predicting the individual vulnerability or resilience to disease is one of the main challenges of modern biomedical research. The question if individual behavioral and physiological characteristics can predict this vulnerability to disease has been subject of debate for a long time (20). Developing strategies to accurately predict the expected degree of disease severity, before onset of disease, is a major challenge for farmers and veterinarians. In our study, we distinguished three types of noninfectious factors that could affect disease outcome. Environmental conditions (housing), individual animal static parameters (coping strategy and sex), and finally parameters may vary in time assessed prior to infection (WBC and lymphocytes, NAb, behavior, rectal temperature, skin lesions, and body weight). These parameters were considered in this study as basic factors available in farm practice. Even though a limited branch of the adaptive immune system was tested in our study (T-cell subsets and granulocytes), many factors of the innate immune system, such as those of the complement cascade and other innate related cytokines and chemokines remain to be examined. Histology lung scores were used as a PRDC read-out measure in order to accurately determine the level of disease development for each pig.

Higher levels of both memory T helper and naïve T helper cells prior to infections were related to lower histology outcome after infection, and this appeared independent of the housing regimen. Under conventional housing conditions only, lower skin lesion scores at the central part of the body, higher levels of KLH binding IgM antibodies, and higher levels of CD8^+^ cells were related to lower histology score after infection.

The majority of predictive models in (veterinary) medicine explains variability in the target outcome by conditioning on observed risk factors of the physical and social environment alone (12). However, these studies do not account for latent sources of variability including individual animal-based variation (21). Proudfoot et al. (12) suggest a flow diagram of the potential linkage between farm management practices and disease risk, including influences from both the social and physical environment. Schulam and Saria (21) proposed a hierarchical model for disease severity prediction previously addressing common latent and observed sources of heterogeneity in complex diseases identifying three levels: the population, subpopulation, and individual levels. According to Schulam and Saria (21), these three levels and possible interactions may possibly reflect biological variation and disease outcome. Our experimental approach can be compared with the flow diagram as suggested by Proudfoot et al. (12), linking individual variation to biological intermediates together with the inclusion of the effect of the physical environment and social stressors. By the provision of the enriched environment to pigs, we improved the possibility for pigs to express their natural behavior in contrast to the conventional housed group. The applied enrichment has proven to reduce stress-related behavior in general and had been demonstrated (13). High stocking densities cause stress by the reduced ability of an individuals to retreat or avoid aggressive behavior from others (22). Another interesting finding in our study was that the conventional housed pigs revealed an increased skin lesion score at the central part of the body after weaning. This was related to more severe histological lung lesions after infection. In most cases, during aggressive mutual fights, the target of biting is at the front third of the body (19). Pigs will get skin lesions at the rump when they try to retreat from the fight (23, 24). This suggests that the animals in our study that were less able to adequately retreat from the fights and subsequently were more prone to develop more severe lung lesions after infection. This was particularly evident under the conventional housing regimen. This relation was not confirmed by the absence of this relation within the enriched housing regimen and absent on the enriched housing environment. A very low skin lesion score was combined with low histology scores in most animals in this group. Skin lesions at the front or rear of the body were not related to histology score, nor were behavioral traits alone. Pre-challenge behavior dynamics [dynamic indicators of resilience (DIORs)] were previously not identified as indicators for PRRSV resilience in terms of morbidity or mortality after infection. Changes in activity levels in early stage of infection were suggested as a useful indictor of resilience in the further trajectory of disease development (25). In this study, the authors especially looked at the dynamics of activity of continuous automated measurements but did not include sex or coping strategy in their analysis. Continuous measurements of behavior allow for more insight into level and dynamics of behavior. Our study observations included the distinctions of play, rooting, interactions with pen-mate aggressive behavior, or other stress-related behaviors, which tentatively influence stress levels and may possibly be missed by automation.

Coping strategies are roughly defined as persistent and are correlated with physiological and behavioral responses of animals to a number of stressors (20, 26). Animals with reactive coping styles are more pronounced hypothalamic–pituitary–adrenal (HPA) responders to social stressors and have been suggested to be at higher risk of infection (12). Our study observations confirmed these findings. We showed that the low resisters' coping strategy was associated with a higher clinical impact of disease in both housing regimens. In addition, other personality traits as well as social status within a group could play a role in clinical outcome.

Others found differences when comparing males and females in their physiological response to infection (27–29). Our findings in which the sex of the animal appeared not to be predictive of the final histology score after infection suggests the contrary. Little evidence has been found to link these sex differences and coping style to disease risk (12). This may be since in general more males are used in research to exclude hormonal cycle influences in females. There are studies in which male pigs were more susceptible to the development of the multifactorial post-weaning multi-systemic wasting syndrome than were female pigs. The authors attributed their findings to castration and associated secondary infections (6).

Our study showed differences in levels of IgG NAb binding KLH and activity prior to infection between males and females. However, these levels of IgG binding KLH or activity did not explain additional variation in disease outcome themselves. Reimert and Rodenburg (30) previously found that enriched housed HR pigs had a higher KLH-IgG titer than enriched housed LR pigs and that both revealed a higher titer than conventional housed HR and LR pigs. These relations were not confirmed in our study.

In general, stressors are thought to have an impact on different parts of the immune system (31). In our study, only limited factors of the immune system were evaluated in which housing regimen itself indeed increased absolute levels of WBC and lymphocytes. These higher levels were related to reduced disease severity as well. No interaction was observed with housing regimen. This suggests that the relation between WBC, lymphocytes, and disease severity applies to both enriched housed pigs and conventional housed pigs.

Temperature, body weight, or growth is frequently proposed to determine disease outcome. In this study, these variables assessed prior to infections were surprisingly not related to severity in clinical outcome after infection. These non-static parameters are probably more of value when measured at the very early stage after infection. Temperature rise, reduced growth rate, and diminished activity are typical symptoms of the disease. Changes in dynamics or level of these parameters can therefore serve as early warning indicators for disease after infection, but not as predictors prior to infections for disease severity.

We additionally tested if prediction in clinical outcome could be improved when using multivariate models. Different variables represent different biological mechanisms and thus they may contribute on top of each other to improve model accuracy. Indeed, accuracy could be slightly improved when adding both absolute counts of memory T helper cells and naïve T helper cells to the model. With regard to the relative levels, also the addition of lymphocytes contributed to model improvement. This multivariate model shows that cell counts of memory T helper and naïve T helper cells (and lymphocytes) prior to infection contribute in addition to each other to the disease severity after infection. The multivariate approach can help to capture dependencies between multivariate trajectories of clinical variables.

This analysis confirms that prediction of disease cannot easily be done using single factors, because complex interactions of external and internal factors exist, and their relations do not always appear to be linear.

We therefore hypothesize that a physiology-based network approach may increase the accuracy of disease severity prediction substantially by including nonlinear relationships. Despite its limited size, this work adds to the body of evidence to explain differences in clinical expression of the polymicrobial diseases observed from pig to pig. This type of challenge studies is useful to establish possible links between external factors, animal-based factors, and clinical outcome of disease. At the same time, they have unavoidable limitations with statistical power and the possibility to extrapolate results to commercial settings. Identifying more complex relations will likely require a larger test group. Nevertheless, this work provides a first basis to unravel the complex network of interactions that will enable a quantitative prediction of disease outcomes in pigs. The methodology presented in this paper serves as a blueprint for identification and quantification of similar networks that are encountered in livestock production.

## Data Availability Statement

The original contributions presented in the study are included in the article/supplementary material, further inquiries can be directed to the corresponding author.

## Ethics Statement

The animal study was reviewed and approved by the Wageningen University Animal Care and Use Committee (Lelystad Department) with number 2013181.

## Author Contributions

ID: design of experiment, conducting experiment, and writing manuscript. DB: statistical analysis writing MM section. JB: interpretation of results and writing discussion. HP: NAb analysis and reviewing manuscript. BK: assisting design of experiment, statistical approach, and reviewing manuscript. SM: statistical approach and reviewing manuscript. NS-Z: phenotyping WBC and pathological assessment and reviewing manuscript. CR: discussing statistical approach and interpretation reviewing paper. JR: writing manuscript and interpretation of results. All authors contributed to the article and approved the submitted version.

## Funding

The study presented in this paper has been funded by the Ministry of Agriculture, Nature and Food Quality, Netherlands (through the knowledgebase project KB-37-001-006).

## Conflict of Interest

The authors declare that the research was conducted in the absence of any commercial or financial relationships that could be construed as a potential conflict of interest.

## Publisher's Note

All claims expressed in this article are solely those of the authors and do not necessarily represent those of their affiliated organizations, or those of the publisher, the editors and the reviewers. Any product that may be evaluated in this article, or claim that may be made by its manufacturer, is not guaranteed or endorsed by the publisher.
