# Corrigendum: Animal-based factors prior to infection predict histological disease outcome in porcine reproductive and respiratory syndrome virus- and *Actinobacillus pleuropneumoniae*-infected pigs

**DOI:** 10.3389/fvets.2024.1350387

**Published:** 2024-02-13

**Authors:** Ingrid D. E. van Dixhoorn, Dennis E. te Beest, Jantina E. Bolhuis, Hendrik K. Parmentier, Bas Kemp, Simon van Mourik, Norbert Stockhofe-Zurwieden, Cornelis G. van Reenen, Johanna M. J. Rebel

**Affiliations:** ^1^Wageningen Livestock Research, Department of Animal Health and Welfare, Wageningen, Netherlands; ^2^Biometris, Wageningen University & Research, Wageningen, Netherlands; ^3^Adaptation Physiology Group, Wageningen University & Research, Wageningen, Netherlands; ^4^Farm Technology Group, Wageningen University & Research, Wageningen, Netherlands; ^5^Wageningen Bio-Veterinary Research, Lelystad, Netherlands

**Keywords:** resilience indicators, porcine respiratory disease, PRRSV, *Actinobacillus pleuropneumoniae*, coping strategy, enriched housing, disease severity, animal-based factors

In the published article, there was an error in Figure 5 as published. The *x*-axis labels in Panel A, “Conventional” and “Enriched,” were placed the wrong way round. The corrected Figure 5 and its caption appear below.

**Figure 5:**
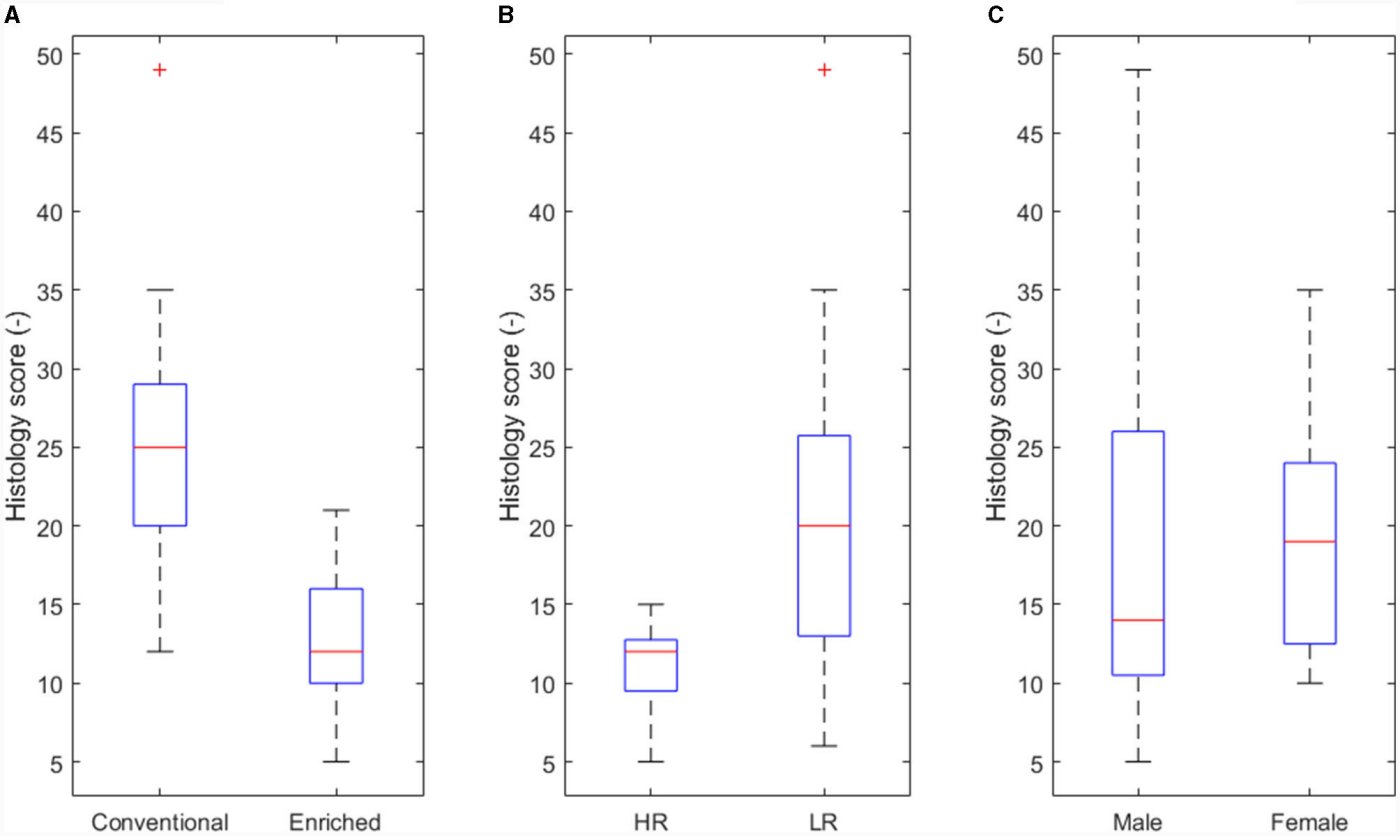
Main effect of experimental variables on histology score. **(A)** Housing (*p* < 0.05). **(B)** Coping strategy (*p* < 0.05). **(C)** Sex not significant (NS).

The authors apologize for this error and state that this does not change the scientific conclusions of the article in any way. The original article has been updated.

